# How perceived risk of recurrence strengthens health management awareness in stroke patients: the chain mediating role of risk fear and health literacy

**DOI:** 10.3389/fpubh.2025.1524492

**Published:** 2025-02-20

**Authors:** Rong Lei, Ming Zhang, Gui Gui, Dajun Yang, Linli He

**Affiliations:** ^1^Health Management Center, Affiliated Hospital of North Sichuan Medical College, Nanchong, Sichuan Province, China; ^2^School of Clinical Medicine, North Sichuan Medical College, NanChong, Sichuan Province, China; ^3^Key Laboratory of Digital-Intelligent Disease Surveillance and Health Governance, North Sichuan Medical College, Nanchong, Sichuan Province, China; ^4^Sichuan Primary Health Research Center, North Sichuan Medical College, NanChong, Sichuan Province, China; ^5^School of Administration, North Sichuan Medical College, Nanchong, Sichuan Province, China; ^6^Institute of Basic Medicine and Forensic Medicine, North Sichuan Medical College, Nanchong, Sichuan Province, China

**Keywords:** stroke patients, perceived risk of recurrence, health management awareness, risk fear, health literacy, health outcomes

## Abstract

**Background:**

Prior research has found that perceived risk in stroke patients motivates health behaviors in visitors. However, the role that perceived risk of recurrence in stroke patients plays in reinforcing health management awareness during the motivation phase is unclear.

**Objective:**

This study explores this issue by examining the effects of risk fear and health literacy on health management awareness due to perceived risk of recurrence in stroke patients.

**Methods:**

We validated the effect of perceived risk of recurrence on health management awareness and its internal mechanism by constructing a structural equation model and including 763 stroke patients, extending the relevant literature and application of the Healthy Behavior Procedural Approach (HAPA) model.

**Result:**

The results suggest that perceived risk of recurrence in stroke patients can effectively reinforce and improve health management awareness, with risk fear and health literacy having a chain-mediated role in this group relationship.

**Conclusion:**

This study reveals the differential effects of perceived risk of recurrence, risk fear, and health literacy in stroke patients on health management awareness at the individual level, providing valuable guidance for healthcare practitioners and families to improve patients’ health outcomes and health well-being.

## 1 Introduction

Stroke is a group of diseases that cause brain tissue damage due to sudden rupture or obstruction of blood vessels in the chest (1, 2). It is the second leading cause of death worldwide (3) and is the third cause of disease affecting disability-adjusted life years (4). The WHO defines stroke as “clinical signs of cerebral dysfunction lasting more than 24 h with no apparent cause other than vascular origin” (5, 6). Strokes have been divided into two main categories: ischemic (80–87%) and hemorrhagic (13–20%) (7, 8). Stroke is the leading cause of death in China in recent years (9), and the burden of stroke in China is the largest in the world, as the number of deaths from stroke accounted for almost one third of the total number of global stroke deaths in 2016 (10). Recurrence is an important risk factor that increases the risk of death and disability from stroke (11). Esenwa and Gutierrez (12) study states that a quarter of all strokes recur and that in the United States, of the 800,000 stroke cases per year, 600,000 are recurrences. In Lekoubou et al. (13) study, patients with recurrent strokes were found to have a 43% higher risk of death than first-time patients. Meanwhile, recurrent ischemic or hemorrhagic damage to brain tissue in patients with recurrent stroke directly affects neurological function, causing cognitive dysfunction and increasing the incidence of dementia (14). Therefore, guiding stroke patients to understand the perceived risk of recurrence and to recognize the seriousness of stroke is of great recurrence significance for improving their treatment outcomes, health behaviors, and health outcomes. However, existing researchers have invested a great deal of time and effort in studying risk factors that cause stroke recurrence, such as cardiovascular-related factors (15) and genetic-metabolic factors (16), lifestyle factors (17, 18). In a recent study, the subjective behaviors of stroke patients (e.g., smoking) were found to be potential risk factors influencing stroke recurrence in the working age group (19). However, this study has not focused on the perceived risk of recurrence of stroke patients, and this neglect is unfortunate because an accurate perception of recurrence risk by stroke patients may change health behaviors and prevent health risks (20). At the same time, numerous studies have shown that most stroke patients do not accurately perceive recurrence risk (21, 22). For example, Croquelois and Bogousslavsky (23) study showed that 65.2% of first-ever stroke patients claimed to be unaware of the risk of recurrence after 3 months. This may diminish stroke patients’ awareness of health management and influence health management behaviors. Therefore, we are concerned that perceived risk of recurrence has important research implications in strengthening health management awareness in stroke patients.

In recent years, health management awareness has gradually become a research hotspot and trend (24, 25). This is closely related to the trend of population aging, changes in health needs, national policy support and health education (26–28). With the continuous extension of the survival cycle of chronic diseases (29, 30), under the influence of population aging and accelerated industrialization, the proportion of chronic disease patient base continues to increase, and the proportion of death due to chronic diseases continues to increase (31). Therefore, it is particularly important to enhance the health management consciousness of patients with chronic diseases. In previous studies, scholars have conducted early explorations, mainly focusing on factors such as illness perception, social support, and psychological resilience (32–34). For example, in the study of Aronoff et al. (35), it was discussed that illness perception and health management are closely related, while social support has also been confirmed to be related to health management in previous studies (36, 37). For example, Cho et al. (38) study, it was found that an individual’s health awareness has a direct impact on the use of health applications. In previous studies, health management awareness involves individuals’ knowledge of health information, assessment of health risks, and proactive actions to improve or maintain their health status (39). For example, in Ahadzadeh et al. (40) study the moderating effect of health management awareness on the perceived usefulness of internet health information and the mediating role of perceived health risks and health information search was found. Although this study has focused on the unique role of health management awareness, it has not explored how health management awareness can be further strengthened. A recent study has proposed that perceived relapse risk has a significant effect on patients’ health behaviors (41). Health behaviors are positive actions driven by health management awareness that contribute to the maintenance and promotion of one’s own health (42, 43). Health Behavior. It remains worth exploring how to further strengthen patients’ awareness of health management and adopt positive and beneficial health behaviors in the context of perceived relapse risk. Specifically, we need to answer the following two questions: does perceived risk of recurrence directly strengthen patients’ awareness of health management? What are the intrinsic mechanisms and boundary conditions by which perceived risk of recurrence affects patients’ health management awareness?

To fill the research gaps mentioned above, we used the Healthy Behavior Procedural Approach (HAPA) model to frame our study. The model divides the behavior change process into two stages: the motivation stage and the action stage (44, 45). Previous research has conducted a large number of studies discussing the influences on health behavior change (46, 47), but few studies have been developed to address the motivation stage. In the motivation stage, individuals form behavioral intentions through the influence of factors such as attitudes, subjective norms, and perceived behavioral control (48). Specifically, the motivational phase of the HAPA model generates attitudes and perceived behavioral control through the interaction of the three cognitive factors of risk perception, outcome expectations, and self-efficacy, which in turn form behavioral intentions (45, 49).

Self-efficacy is a key factor in the motivational phase of the HAPA model. The health literacy of patients is able to assess health risks, form positive outcome expectations and sense of self-efficacy, and thus be more likely to adopt and maintain healthy behaviors (50, 51). This is mainly due to the fact that individuals with high health literacy are better able to formulate and execute plans and thus more effectively translate behavioral intentions into actual behaviors (52). Specifically, in chronic disease management, patients with high health literacy are more likely to follow medical advice and have regular health examinations and treatments. Therefore, patients with higher health literacy can better understand the recurrence risk information, evaluate and manage their health behaviors more effectively, so as to enhance the influence of risk fear on health behavior intention. At the same time, risk fear, as the expressed fear of risk perception, refers to the individual’s worry and fear of disease recurrence (53). This fear can serve as a motivational factor to prompt patients to pay more attention to their health status and motivate them to take preventive measures. According to the HAPA model, risk fear affects individuals’ health management intention by affecting their attitudes and subjective norms. When patients are fearful of relapse risk, they are more likely to think it is beneficial to adopt healthy behaviors and strengthen health management awareness driven by health literacy. Therefore, in the planned behavior model, health literacy and risk fear may act as chain mediating variables to affect the relationship between stroke patients’ perception of recurrence risk and health management awareness. Specifically, individuals with high health literacy are more likely to accurately understand relapse risk information and evaluate and manage their health behaviors more effectively, thus enhancing the influence of risk fear on health behavior intentions.

This paper presents a new perspective and theoretical framework in the field of stroke research with significant novelty. Firstly, this article focuses on the importance of perceived recurrence risk and health management awareness, and further discusses how to strengthen patients’ health management awareness through perceived recurrence risk, which provides new theoretical support for this field. Second, this paper uses the HAPA model to construct a research framework to explore the key role of health literacy in the motivation stage. It is proposed that individuals with high health literacy are better able to formulate and execute plans, thereby more effectively transforming behavioral intention into actual behavior. Third, this paper proposes health literacy and risk fear as chain mediating variables, and explores their role in the relationship between stroke patients’ perception of recurrence risk and health management awareness. This provides a new perspective for understanding how patients enhance health behavior intention through health literacy and risk fear. Therefore, this paper provides new insights into the field of stroke research by systematically exploring the risk perception of stroke recurrence, health management awareness, the application of the HAPA model, and the chain mediating effect of health literacy and risk fear.

## 2 Literature review and theoretical derivation

### 2.1 Perceived risk of recurrence and health management awareness

Perceived risk of recurrence refers to the patient’s perception of the probability of recurrence risk as well as its severity (54). It involves the patient’s perceived level of recognition of warning symptoms, likelihood of recurrence, and severity of the consequences of recurrence (55). In existing studies, it has been shown that stroke patients’ perception of recurrence risk tends to be lower than the actual risk, which may lead to a behavior ingress of patients to adopt preventive behaviors (21, 22). Therefore, it is particularly important to pay attention to the factors that influence the perceived risk of recurrence in stroke patients, such as personal characteristics, their own illness, family factors, and healthcare support (56–58). Therefore, it is particularly important to increase patients’ attention to the perceived risk of relapse.

Health management awareness refers to an individual’s self-consciousness about his or her health status and maintenance, as well as the awareness of taking positive measures to prevent disease and maintain and promote health (59). Patients with a high level of health consciousness are concerned about disease treatment and will place more emphasis on lifestyle modification and the development of healthy habits (60, 61). However, in life, the awareness of health management among stroke patients faces multiple challenges, on the one hand, there are deficiencies in chronic disease service management and service models (62). The growth of health service consumption and increased consumer demand for chronic disease management promotes the need for health management service providers to continuously innovate their service models. On the other hand, the popularity and acceptance of health management services have yet to be improved (63, 64). For example, although the importance of regular medical checkups is widely recognized, not all people have regular health checkups (65). Therefore, few studies have explored the emerging antecedents of health management awareness from the perspective of patients themselves. Our study focused on patients’ perceived risk of recurrence as an important influence on patients’ health management awareness. Based on the motivational stage of the HAPA model, we propose that in stroke patients, perceived recurrence risk affects the individual’s attitude and perceived control, which in turn affects their behavioral intention and actual health behavior. Specifically, when patients perceive a higher risk of recurrence, they may place more emphasis on health management and thus take more proactive preventive measures. However, if patients perceive a low risk of recurrence, this may lead them to neglect health management, thereby increasing the risk of recurrence.

Based on this, we propose the following hypothesis 1:

*H1*: Perceived risk of recurrence in stroke patients can effectively reinforce health management awareness.

### 2.2 The mediating role of health literacy

Health literacy refers to an individual’s ability to access, understand, and process basic health information or services that help individuals make good decisions to maintain and promote their health (66, 67). The A reading of the literature reveals that a large number of existing studies have focused on two aspects of health literacy, one being the importance of health literacy in promoting the formation of healthy behaviors and lifestyles, the prevention of diseases, and the improvement of population health (68, 69). For example, in Zwierczyk et al. (70), which discusses health information literacy to promote the adoption of healthy eating habits among rural residents; on the other hand, research on health literacy in healthcare settings centered around physicians and patients, which explores the improvement of physicians’ ability to quickly identify patients with insufficient health literacy in stressful and busy healthcare environments, and to deliver health information to patients more effectively, as well as patients’ ability to access, understand, and accept health information (71–73). In recent years, health literacy research has gradually focused on multidisciplinary cross-cutting areas (74, 75) For example, researchers have emphasized the integration of clinical medicine and preventive medicine to make the study of health literacy-related issues more comprehensive (76). For example, in the For example, in Tavakoly Sany et al. (77), it was found that physicians’ communication skills can improve patients’ self-efficacy, enhance patient compliance, and improve treatment outcomes. However, this study never talked about how health literacy can strengthen patients’ awareness of health management in the context of stroke patients’ perceived risk of recurrence.

Stroke patients have varying degrees of cognitive dysfunction, somatic mobility disorders, speech disorders, and many other types of dysfunction after the onset of stroke (78, 79). At the same time, stroke patients suffer from impaired perceptual-perceptual functioning after the onset of the disease, with reduced ability to receive and understand information (80, 81). This reduces the ability of patients to receive and understand information. This reduces the patient’s ability to perceive the risk of recurrence to some extent. This often leads to worse health outcomes as patients are unable to better acquire knowledge about stroke prevention and management, and are unable to effectively process and understand medical information. Therefore, it is particularly important to strengthen health literacy among stroke patients. Health literacy serves as a key cognitive factor where patients are able to access, understand, and process basic health information or services (82, 83). Specifically, patients with high health literacy are more likely to follow a treatment regimen that includes long-term medication, regular follow-up appointments, and implementation of lifestyle changes (84). Additionally, individuals with high health literacy are better able to identify and understand health risk factors (84). This awareness enables them to take proactive preventive measures such as regular medical checkups, a healthy diet, and moderate exercise. For example, stroke patients with high health literacy will be more likely to recognize the impact of chronic conditions such as hypertension and diabetes on their health and actively manage these chronic conditions to reduce the risk of stroke recurrence (85). It can be seen that health literacy strengthens stroke patients’ ability to critically analyze health information, enabling them to identify and avoid inaccurate or misleading health information, and thus make more informed health decisions.

Based on this, we propose the following hypothesis H2:

*H2*: Health literacy mediates perceived risk of recurrence and health management awareness in stroke patients

### 2.3 The mediating role of fear of risk

Risk fear is the tendency of individuals to express behaviors that avoid or reduce risk in the face of potential loss or uncertainty (86). Individuals with risk fear tend to prefer certainty of outcome, even if that certainty is lower in expected return value than the risky option. For example, risk-averse investors may prefer low-risk, low-return investments to high-risk, high-return investments (87). However, in real life, the degree and behavior of individual risk fear are often influenced by an individual’s personality traits, cultural background, experience, and emotional state (88, 89). Thus, risk fear may lead to overly conservative decision-making, or it may be a protective mechanism against potential negative consequences. In previous studies, fear of risk has been focused on safety management, individual differences, decision-making errors, and mental health (90). For example, Alsolais et al. (91) et al. focused on how fear of risk affects individuals’ levels of anxiety and depression. However, previous studies have not focused on the effects of fear of risk on stroke patients. The severe consequences and unpredictability of stroke as a sudden onset brain disease make patients susceptible to fear of disease progression (92, 93). This fear can affect patients’ emotional state, treatment adherence, and the rehabilitation process and may even lead to avoidance behaviors such as reduced activity or refusal to participate in rehabilitation training, affecting their quality of life and health outcomes (94, 95). Therefore, it is important that we study the risk fears of stroke patients.

Fear of risk often leads to avoidance behavior in patients (96). For example, stroke patients reduce regulatory activities due to fear, which not only limits the patient’s ability to perform activities of daily living but may also increase the risk of falls, creating a vicious circle (97, 98). The risk of falling may also increase the risk of falling. At the same time, fear of risk may lead to a decrease in patients’ perceptions of the disease and self-efficacy, as well as the development of adverse emotions such as fear and anxiety (99, 100). Such emotions weaken patients’ health management awareness and behavior. According to Guglielmi et al. (101) study, patients have difficulty perceiving the risk of recurrence of stroke disease in relation to stroke disease. However, perceived risk of recurrence in stroke patients is an important influencing factor in causing risk fear in patients. Specifically, patients with high perceived risk of recurrence are more likely to have high-risk fear, causing patients to potentially fall into a prolonged state of stressful stress, whereas patients with low perceived risk of recurrence tend to have lower-risk fear, increasing patients’ willingness to cooperate with treatment. However, we found through the literature that although previous studies have focused on the correlation between risk fear and perceived risk of recurrence, they have not focused on health management awareness among stroke patients. Specifically, previous studies have failed to focus on risk fear mediating the relationship between perceived risk of recurrence and health management awareness in stroke patients. This prediction has never been confirmed. However, some indicative evidence does currently exist regarding patients’ health management awareness and risk fear. For example, in Chen et al. (102) study, fear was found to be an important influence on the formation and transformation of patients’ health management awareness and behavior. The theme of our study is that perceived risk of recurrence in stroke patients may enhance patients’ health management awareness by increasing risk fear.

This is because risk fear is a strong emotional response conveyed by patients gathering medical information (103). In particular, the ability to gather medical information is a specific manifestation of patient health literacy (66). We believe that patients with high health literacy We suggest that patients with higher health literacy are more likely to accurately understand medical information, recognize early symptoms of recurrence, and take preventive measures, thereby reducing the perceived risk of recurrence. Further, health literacy not only affects patients’ perceived assessment of recurrence risk but may also influence their behavioral decisions by influencing their level of risk fear. When patients have a higher perceived risk of disease recurrence, they may develop a stronger fear of risk, and this fear motivates them to pay more attention to health management and further strengthens their awareness of health management. At the same time, improved health literacy helps patients more accurately assess risk, reduce unnecessary fear, and develop a positive sense of health management. Therefore, health literacy and risk fear work together to influence the perceived risk of recurrence and health management awareness of stroke patients.

Based on this, we propose the following hypothesis:

*H3*: The mediating role of fear of risk on perceived risk of recurrence and health management awareness in stroke patients.

*H4*: Health Literacy and Fear of Risk Chain-Mediate Perceived risk of recurrence and Health Management Awareness in Stroke Patients.

**Figure 1:**
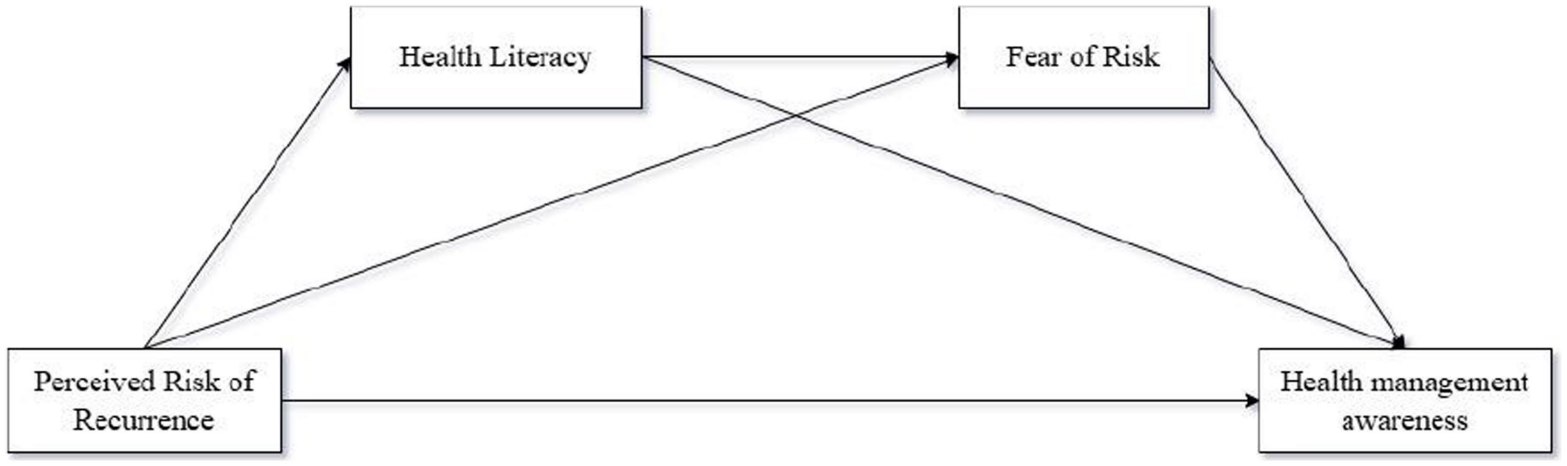
Framework diagram of the theoretical model.

The theoretical model is shown in Figure 1.

## 3 Methods

### 3.1 Sample sources

This study examined how perceived risk of recurrence strengthens health management awareness in stroke patients. Therefore, our target participant inclusion criteria were (1) clinically diagnosed stroke patients, (2) with average verbal communication skills, and (3) without significant cognitive impairment. Among them, we used the Mini-Mental State Examination Scale to test cognitive impairment, with the criterion of a total score less than 23 points (104, 105).

This study was approved by the Academic Ethics Committee of North Sichuan Medical College. Stroke patients were from the patients in the Affiliated Hospital of North Sichuan Medical College. After obtaining the consent of the patients and their families, we went to the family of the patients for data collection. All participants signed an electronic informed consent form, described in the approval document of the Academic Ethics Committee of North Sichuan Medical College. Parental informed consent was not required as this study did not involve patients under 18.

The data collection period was from December 2024 to January 2025, and 700 questionnaires were distributed. The questionnaires with strong consistency and short response time (less than 2 min) were excluded, and 64 samples were excluded. Of these, the sample consisted of 405 male subjects and 231 female subjects. Seventy participants aged 18 to 25; 110 participants aged 26–40 years; 146 participants aged 41 to 60 years; 188 participants aged 61–70 years; there were 122 participants aged 71 years. See Table 1 for specific demographic information.

**Table 1:** Summary of demographic information.

Variant	Items	Frequency	Percentage
Gender	Male	405	61.2%
	Female	231	34.9%
Age	18–25 years	70	11%
	26–40 years	110	17.3%
	41–60 years	146	23%
	61–70 years	188	29.6%
	71 and over	122	19.2%
Education background	Primary school	107	16.8%
	junior high school	149	23.4%
	Technical secondary school, high school	186	29.2%
	Junior college	120	18.9%
	Undergraduate college	71	11.2%
	Postgraduate	1	0.2%
	Doctoral student	1	0.2%
Place of residence	Urban	247	38.8%
	Suburban	389	61.2%
Marital status	Married	389	61.2%
	Unmarried	99	15.6%
	Divorced	114	17.9%
	Widowed	34	5.3%
Monthly income level (RMB)	2000 and below	26	4.1%
	2001–3,000	327	51.4%
	3,001–4,000	246	38.7%
	4,001–5,000	26	4.1%
	5,001 and above	11	1.7%

### 3.2 Research methodology

#### 3.2.1 Perceived risk of recurrence scale

The perceived risk of recurrence scale is referenced in Jin et al. (106). Our measurement questions were adapted from the second part of the scale, which consists of three dimensions with a total of 14 items. The three dimensions are perceived relapse severity, perceived disease risk factors, and perceived behavioral risk factors. We scored according to a 5-point Likert scale. Higher scores indicate a higher perceived risk of recurrence for the patient. In this study, the Cronbach’s alpha for this scale was 0.93. Referring to Hayduk (107) criteria, the canonical chi-square value (CMID/DF = 7.281) of the model in this study performed excellently. Referring to Bagozzi and Yi (108) and Scott and Bruce (109) criteria, the overall fit index (GFI = 0.89), adjusted fit index (AGFI = 0.844), comparative fit index (CFI = 0.924), corrected fit index (TLI = 0.907), and root-mean-square-error approximation of fit (RMSEA = 0.099) of the model of the present study are of excellent fit.

#### 3.2.2 Health literacy scale

The Health Literacy Scale is referenced in Chinn and McCarthy (110). Our measurement questions were adapted from the three dimensions of the scale, totalling seven items. The three dimensions are functional health literacy, communicative health literacy, and critical health literacy. In this study, the Cronbach’s alpha for this scale was 0.892. Referring to Hayduk (107), Bagozzi and Yi (108), Scott and Bruce (109), the criterion of validated factor analysis (CFA) produced metrics indicating acceptable model fit: CMID/DF = 6.153, GFI = 0.977, AGFI = 0.921, CFI = 0.982, TLI = 0.954, RMSEA = 0.09. The model was well fitted.

#### 3.2.3 Risk fear scale

The fear of risk scale is referenced in Champion et al. (111) on the Breast Cancer Fear Scale, and our questions were adapted from the 10 questions of this scale. For example: Do you agree that you feel afraid when you think about the serious consequences of a recurrent stroke? In our study, the Cronbach’s alpha for this scale was 0.777. Referring to Hayduk (107), Bagozzi and Yi (108) and Scott and Bruce (109), the criteria of the validated factor analysis (CFA) produced metrics indicating acceptable model fit: CMID/DF = 6.291, GFI = 0.959, AGFI = 0.896, CFI = 0.962, TLI = 0.923, RMSEA = 0.091, good model fit.

#### 3.2.4 Health management awareness scale

The Health Management Awareness Scale was selected with reference to the work of Kim et al. (59) study. Compared with Kim et al. (59), we chose the Health Management Awareness Scale (HMAS). Ware (112) ‘s Perceived Health Scale, which used five variables, including mental health, disease manaccd4gement, sleep management, diet management, and hygiene management, totaling 17 measurement questions. In our study, the Cronbach’s alpha for this scale was 0.843. Referring to Hayduk (107), Bagozzi and Yi (108) and Scott and Bruce (109), the criteria, the validated factor analysis (CFA) produced metrics indicating acceptable model fit: CMID/DF = 3.961, GFI = 0.925, AGFI = 0.894, CFI = 0.96, TLI = 0.95, RMSEA = 0.068. The model was well fitted.

## 4 Results

### 4.1 Common methodological deviations

Referring Liu et al. (113) study, we used Harman one-way analysis of variance to test the common method bias problem by subjecting all the variables to exploratory factor analysis. The results found that: a total of 9 factors with Eigen roots greater than 1 appeared in the unrotated case, and the explanation rate of the first factor was 33.04%, which did not exceed the critical value of 40%, indicating that there was no serious common method bias in this study.

### 4.2 Normality test

Normality tests for each variable were performed using skewness and kurtosis. According to the normality test standard proposed by Kline (114): the absolute value of skewness coefficient is less than 3, and the absolute value of kurtosis coefficient is less than 8, the sample data can be considered to meet the requirements of approximately normal distribution. According to the analysis results in Table 2, the absolute values of kurtosis and skewness of the measurement items of each variable in this study are within the standard range. Therefore, the measured data of each variable satisfied the approximate normal distribution.

**Table 2:** Results of normality tests for variables.

Variable	M	SD	Skewness	Kurtosis
Perceived risk of recurrence	2.345	0.627	−0.117	0.333
Health literacy	2.636	0.674	−0.267	0.315
Fear of risk	3.024	0.554	−1.069	2.533
Health management awareness	3.281	0.506	−1.178	2.956

### 4.3 Correlation analysis

We performed descriptive statistics and correlation analysis of perceived risk of recurrence, health literacy, risk fear and health management awareness. The results showed that: perceived risk of recurrence was significantly and positively correlated with health literacy (*r* = 0.66, *p* < 0.01); perceived risk of recurrence was significantly and positively correlated with fear of risk (*r* = 0.365, *p* < 0.01); perceived risk of recurrence was positively and positively correlated with awareness of health management (*r* = 0.429, *p* < 0.01); and health literacy was positively correlated with fear of risk (*r* = 0.463, *p* < 0.01). Health literacy and health management awareness had a positive correlation (*r* = 0.491, *p* < 0.01); and risk fear and health management awareness had a positive correlation (*r* = 0.515, *p* < 0.01). See Table 3 for details.

**Table 3:** Descriptive statistics and correlation analysis results for each variable.

Variant	M	SD	1	2	3	4
1. Perceived risk of recurrence	2.345	0.627	1			
2. Health literacy	2.636	0.674	0.66**	1		
3. Fear of risk	3.024	0.554	0.365**	0.463**	1	
4. Health management awareness	3.281	0.506	0.429**	0.491**	0.515**	1

### 4.4 Tests of the mediating effect of health literacy

**Figure 2:**
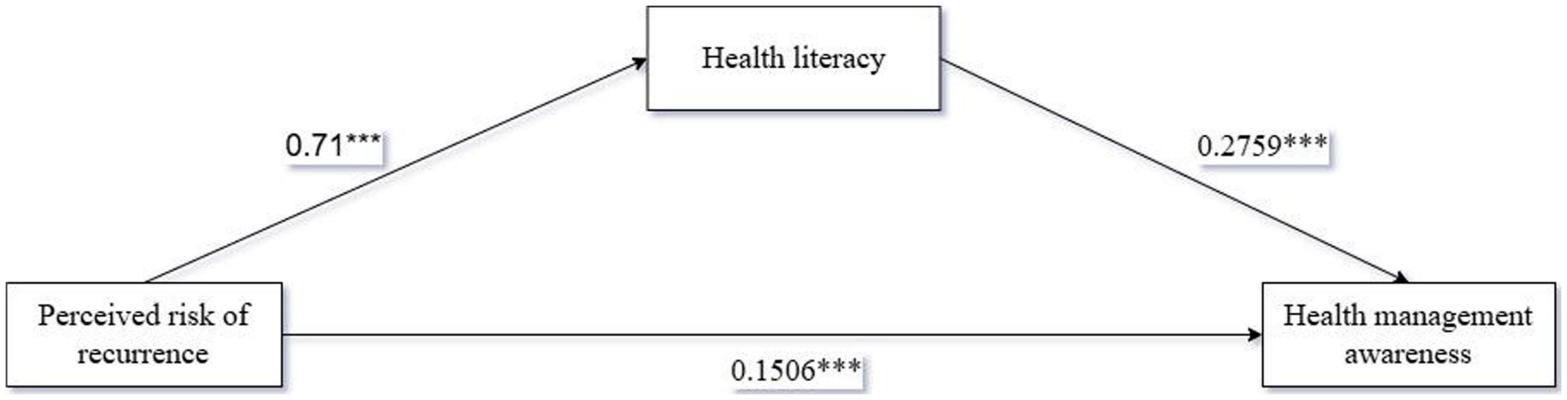
Path coefficients of the mediating effect of health literacy.

We used health literacy as the mediating variable, perceived risk of recurrence as the independent variable, and health management awareness as the dependent variable. We used Process Model 4 to analyze the mediating relationship between health literacy for perceived risk of recurrence and health management awareness. [Bootstrap sample: 5000, (90)]. The results showed that the mediation process of perceived risk of recurrence-health literacy-health management awareness was significant [*β* = 0.1959, SE = 0.0278, 95%CI (0.1427,0.2523)]. The coefficient of perceived risk of recurrence-health management awareness was 0.1506***; the coefficient of perceived risk of recurrence-health literacy was 0.71***; and the coefficient of health literacy-health management awareness was 0.2759***. Therefore, health literacy is a perfect mediator between perceived risk of recurrence and health management awareness as shown in Figure 2, testing hypothesis H2.

### 4.5 Tests of the mediating effect of fear of risk

**Figure 3:**
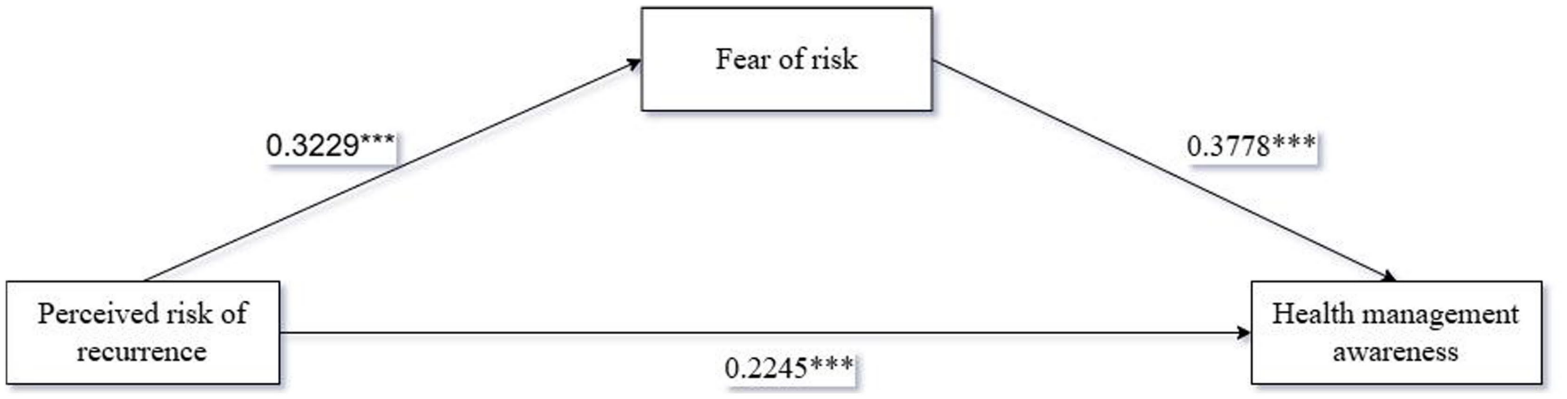
Path coefficients of the mediating effect of fear of risk.

Immediately after that, we analyzed the mediating relationship of risk fear on perceived risk of recurrence and health management awareness using Process Model 4 with risk fear as a mediating variable [Bootstrap sample: 5000, (90)]. The results showed that: perceived risk of recurrence had a significant effect on health management awareness (*β* = 0.2245, *p* < 0.001); perceived risk of recurrence had a significant effect on fear of risk (*β* = 0.3229, *p* < 0.001); and fear of risk had a significant effect on health management awareness (*β* = 0.3778, *p* < 0.001). Overall, the mediation process of perceived risk of recurrence-risk fear-health management awareness was significant [*β* = 0.122, SE = 0.0234, 95%CI (0.0803,0.1722)]. Therefore, fear of risk was fully mediated between perceived risk of recurrence and health management awareness, as shown in Figure 3, testing hypothesis H3.

### 4.6 Chain mediation effect test for health literacy and fear of risk

**Figure 4:**
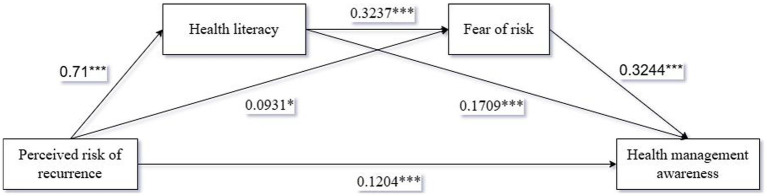
Path coefficient plots of the chain-mediated effects of health literacy and fear of risk.

Finally, we used Process Model 6 to analyze the chain mediation of health literacy and fear of risk by using both health literacy and fear of risk as mediating variables [Bootstrap sample: 5000, (90)]. The results showed that: the chain mediating effects of health literacy and risk fear on perceived risk of recurrence and health management awareness were fully significant [*β* = 0.0745, SE = 0.0158, 95%CI (0.0464,0.1085)]. Among them, health literacy mediated significantly between perceived risk of recurrence and health management awareness [*β* = 0.1213, SE = 0.0273, 95%CI (0.0685,0.1769)]; risk fear mediated significantly between perceived risk of recurrence and health management awareness [*β* = 0.0302, SE = 0.0151, 95%CI (0.0038,0.0623)]. Perceived risk of recurrence had a significant effect on health management awareness (*β* = 0.1204, *p* < 0.001); perceived risk of recurrence had a significant effect on fear of risk (*β* = 0.0931, *p* < 0.05); fear of risk had a significant effect on health management awareness (*β* = 0.3244, *p* < 0.001); Health literacy had a significant positive effect on fear of risk has a significant positive effect (*β* = 0.3237, *p* < 0.001); the coefficient of perceived risk of recurrence on health literacy is 0.71***; the coefficient of health literacy on health management awareness is 0.1709***. Therefore, the chain mediating effects of health literacy and fear of risk on perceived risk of recurrence and health management awareness were fully significant as shown in Figure 4, which verified hypothesis H4.

## 5 Discussion and conclusions

### 5.1 Theoretical contributions

This study has several important theoretical implications.

First, in past studies, patients’ risk perception has been identified as influencing patient health behavior (66, 115). However, few studies have focused on patients’ perceived risk of recurrence and explored how perceived risk of recurrence in stroke patients affects patient health motivation. Although patients’ perceived risk of recurrence has been recognized as one of the important factors influencing patients’ health awareness, its impact on patients’ health management awareness has not been fully explored. Therefore, this study responds to the academic interest in a research area that emphasizes the role of perceived risk of recurrence in motivating health management in stroke patients. By integrating these factors into the health management of stroke patients, this study extends the relevant literature and deepens the understanding of patients’ awareness of health management. This study enriches the literature on the relationship between perceived risk of recurrence and health management awareness in stroke patients; on the other hand, this study extends existing knowledge by revealing new antecedent factors and provides a better understanding of how perceived risk of recurrence enhances patients’ health management awareness.

Second, based on the motivation theory of the HAPA model, this study examined the mediating role of risk fear from recurrence perceived risk and patients’ own health literacy in stroke patients. We revealed the mechanism of the mediating role of recurrence perceived risk on stroke patients’ health management awareness through risk fear and health literacy from the motivational stage of the HAPA model. Our study extends existing research on the association between patients’ risk perception and health behaviors, such as mortality risk perception and healthy rest and relaxation, healthy diet, disease management, and so on (116–118). However, few studies have discussed how risk perception strengthens patients’ awareness of health management. This finding not only enriches the application of HAPA modeling in the field of chronic disease management, but also provides a new theoretical perspective on health management of stroke patients. We identified and intervened in key psychological factors affecting stroke patients’ health management awareness by constructing a structural equation model to promote patients’ health behavior change and reduce the risk of recurrence. The importance of improving the health literacy of stroke patients is emphasized in this process, providing a theoretical basis for developing targeted health education and interventions.

Then, the difference in the influence of stroke patients’ perceived recurrence risk on health literacy and risk fear is mainly due to the dual mechanism of cognition and emotion. From the perspective of cognition, the improvement of health literacy depends on the individual’s ability to understand, evaluate and apply health information. When stroke patients perceive the risk of recurrence, they will actively seek related health knowledge to better understand the disease and master prevention and management methods, such as actively asking medical staff about the factors of disease recurrence and preventive measures, so as to improve health literacy. At the same time, recurrence risk perception may also stimulate patients’ critical thinking on health information, make them pay more attention to the accuracy and reliability of information, and improve their ability to evaluate health information. From the emotional level, risk fear, as a direct and strong emotional response, plays an important role in the perception of recurrence risk. When patients realize that they may face disease recurrence, their fear is quickly awakened. This fear not only stems from the worry about the pain and harm caused by the disease itself, but also may include the decline of quality of life after recurrence and the aggravation of family burden. This strong emotional experience prompts patients to have a strong motivation to take action to avoid or reduce the occurrence of risks, thus significantly affecting their behavioral decisions and the execution of health behaviors in the short term.

Finally, this study enriches the existing literature on the relationship between health literacy and risk fear, especially in the area of major public health events and major diseases. In previous studies, patients’ fear of risk has been mostly associated with public health events, whereas research on the fear of risk associated with major recurrent diseases is still in its early stages. Influential factors regarding risk fear are mainly derived from patients’ cognitive trust, social support, and fall experiences (119, 120). Few studies have focused on patients’ subjective factors, such as patients’ emotions, and personality traits. The present study examined the impact of patients’ health literacy as a subjective factor on fear of risk. This not only remedies the neglect of previous studies on patients’ risk fear and its impact on subjective factors, but also calls for future studies to focus on patients’ risk fear in the face of major recurrent diseases.

### 5.2 Implications for practice

Health management of stroke patients is a complex and multidimensional issue in both the psychological and medical fields, and it is particularly important to strengthen the health management awareness of stroke patients. Focusing on risk fear and health literacy triggered by perceived risk of recurrence in stroke patients, this study aims to reveal how these factors jointly influence should patients’ health management awareness and long-term health outcomes. This provides a framework for gaining insights into the health management awareness and psychological state of stroke patients, which can help develop more precise and effective interventions.

By first exploring the relationship between perceived risk of being relapsed and fear of risk, this study allows healthcare professionals to gain a deeper understanding of patients’ possible avoidance behaviors and emotional responses. This understanding is critical to the development of an individualized recovery plan. This understanding allows professionals to anticipate and address psychological barriers that may impede the recovery process. For example, if a patient avoids necessary physical therapy due to fear of relapse, professionals can design interventions to alleviate this fear, thereby improving treatment adherence and outcomes. In addition, this study explores the mediating role of health literacy, revealing how patients utilize health information to manage their illness. Patients with higher health literacy are more likely to understand the risk of recurrence and take appropriate preventive measures, such as improving lifestyle, following medical advice, and regularly monitoring health status. Therefore, improving the health literacy of the stroke patient population is key to improving their ability to self-manage and reduce the risk of recurrence.

Immediately following, this study provides a basic guideline for improved long-term health outcomes for patients. In this study, we demonstrated that fear of risk enhances health management awareness in stroke patients. Consequently, we believe that reducing fear of risk can improve patients’ quality of life. Fear and anxiety associated with the disease not only affect patients’ emotional state, but also limit their social activities and daily functioning. Effective psychological interventions by healthcare workers or family members can help stroke patients better manage these negative emotions, thereby enhancing their overall well-being and improving health outcomes. In addition, improving patients’ health literacy is another key way to improve health outcomes. Improved health literacy helps patients make more informed health decisions, as evidenced by the patient’s ability to understand, evaluate, and apply medical information. Healthcare workers or family members improve the health literacy of stroke patients through health education or health promotion activities so that patients can better understand their condition, follow their treatment plan more effectively, and seek medical help when necessary. For example, medical workers and community workers can carry out health popularization of stroke knowledge, improve the public’s understanding of the cause of stroke, treatment methods, nursing methods and other knowledge, and improve the participation of the public.

The motivation theory of HAPA model not only plays an important role in the health management of stroke patients, but its theoretical framework and application methods also have wide applicability and extrapolation, which can provide effective guidance and support for health behavior change in other patient groups and different environments. For example, the motivational theory of the HAPA model is able to provide scientific theoretical guidance for rehabilitation promotion programs. For patients with sequelae of stroke and other groups who need long-term rehabilitation, patients should understand the risks of disease recurrence and limited functional recovery if they interrupt treatment or do not adhere to rehabilitation training during the rehabilitation process, so as to stimulate their fear of rehabilitation interruption. At the same time, the health literacy of patients on rehabilitation training, drug therapy and other health behaviors should be improved to make them realize the important role of these behaviors in promoting functional recovery and improving the quality of life. On this basis, a detailed rehabilitation plan was made, and professional rehabilitation therapists were arranged for guidance to help patients adhere to the implementation of rehabilitation behavior in the action stage and enhance their awareness of health management in the rehabilitation process.

Risk fear is a complex psychological response, which can come from the patient’s perception of the risk of recurrence, or it can lead to excessive anxiety, which has a negative impact on the patient’s recovery. The community can help patients understand the risk of stroke recurrence more comprehensively by carrying out regular health education activities, so as to improve their awareness of health management. However, fear can also trigger excessive anxiety, so psychological support services should also be provided in the community to help patients ease their psychological burden. In clinical practice, fear can promote stroke patients to pay more attention to their own health, so as to improve the perception of recurrence risk. This enhanced perception helps patients to actively take health management measures, such as regular medication, regular physical examination, and improvement of lifestyle. On the other hand, excessive fear may cause patients to fall into anxiety and helplessness, and even affect their compliance with treatment. Therefore, clinicians need to pay close attention to the patient’s psychological state during the treatment process, and timely identify and intervene in the negative emotions caused by fear. Cognitive behavioral therapy, psychological counseling and patient education are used to help patients establish correct risk cognition, relieve unnecessary fear, and improve patients’ self-management ability and treatment compliance.

### 5.3 Limitations and future research

Despite the above findings, this study still has some flaws. This study was a single-center study and may not be representative of all stroke patient populations. Therefore, we suggest that future studies may expand the data sources to include studies from multiple centers, different regions, and different populations to enhance the accuracy and reliability of the results. Second, this study mainly explored the mediating roles of perceived risk of recurrence and health management awareness in stroke patients, and has not yet delved into the possible moderating effects between the two. Therefore, in future studies, we suggest that researchers can add moderating variables and continue to explore the internal mechanisms and boundary moderation of perceived risk of recurrence and health management awareness in stroke patients to enhance the scientific and explanatory power of the model. Finally, this study serves as a preliminary exploration of health management awareness in stroke patients, and we ask several interesting questions, such as: what is the correlation between the perceived risk of recurrence in stroke patients and patients’ personality traits? How does health management awareness in stroke patients motivate patients to adopt healthy behaviors? These questions can be answered in future studies.

## 6 Conclusion

This study demonstrated the relationship between perceived risk of recurrence and health management awareness among stroke patients and its internal mechanisms by constructing structural equation modeling. Specifically, there is a complex chain-mediated relationship between perceived risk of recurrence and health management awareness, in which patients’ risk fear and health literacy are important influencing variables. This model can enrich the content of Health Behavior Procedural Model and related theories from the perspective of behavioral change motivation, and provide new ideas for the study of health management awareness. Given the significant impact of risk fear on health management awareness, psychological interventions are recommended to be introduced into clinical care. For example, cognitive behavioral therapy can help patients ease fear-driven anxiety and increase confidence in coping with relapses. At the community level, it is recommended to promote the chronic disease management model based on the concept of “active health.” To improve the health literacy and self-management ability of patients through community health education and digital health intervention tools. For example, developing easy-to-use health management applications that help patients monitor health indicators in real time and provide personalized health recommendations.

## Data Availability

The original contributions presented in the study are included in the article/supplementary material, further inquiries can be directed to the corresponding authors.
